# Psychological distress, chronic conditions and quality of life in elderly hematologic cancer patients: study protocol of a prospective study

**DOI:** 10.1186/s12885-017-3662-1

**Published:** 2017-10-25

**Authors:** Norbert Köhler, Anja Mehnert, Heide Götze

**Affiliations:** Department of Medical Psychology and Medical Sociology, University Medical Center Leipzig, Philipp-Rosenthal-Strasse 55, 04103 Leipzig, Germany

**Keywords:** Hematologic malignancies, Aged, Quality-of-life, Psychological distress, Chronic conditions

## Abstract

**Background:**

Similar to most solid tumors, the incidence of hematologic malignancies has been rising. Although the median age at diagnosis is about 70 years, little is known about psychosocial aspects and comorbid conditions in elderly patients with hematologic cancers. The main objectives of our study are to assess the prevalence of psychological distress, chronic conditions, functional disabilities, and quality of life in both elderly hematologic cancer patients aged ≥70 years and an age-matched comparison sample of the general population.

**Methods:**

We conduct a prospective study with three measuring points (t_1: ≥5 years after first time hematologic cancer diagnosis / relapse; t_2 and t_3 six months and 1 year after t_1). In addition, we use a cross sectional study design to recruit a comparison sample of the general population matched by age and sex. Both samples, patients and the comparison group complete validated questionnaires measuring psychological distress, chronic conditions, functional disabilities, and quality of life as well as health care needs and health care utilization.

**Discussion:**

Our study will provide both a data set offering detailed information about elderly hematologic cancer patients’ physical, psychological and demographic characteristics, and reference data of the elderly general population. Furthermore, the study will provide important information for the development and implementation of psychooncological support offers and survivorship care plans.

## Background

### Hematologic malignancies

Hematologic malignancies comprise a heterogeneous spectrum of neoplasms including leukemia, lymphoma, and plasma cell neoplasms. Similar to other malignancies, the overall incidence of hematologic cancer appears to be rising [1, 2]. Taken together, hematologic malignancies account for 8% of new cancer diagnoses. While the first peak of incidence of hematologic malignancies is found in childhood and adolescence (esp. acute lymphocytic leukemia), the second peak of incidence is observed in the old age [3].

Since the spectrum of hematologic malignancies is rather heterogeneous, hematologic cancer therapies depend on the kind of neoplasm [4]. Treatment for aggressive hematologic cancers often goes along with multiple periods of intensive inpatient treatment and debilitating side effects [5]. Patients undergoing bone-marrow or stem-cell transplantation are confronted with a range of severe adverse events, including conditioning (e.g. destruction of endogenous immune cells), immunosuppressive medication and life threatening immune reactions (e.g. Graft-versus-Host-Disease) [6]. More chronic forms of hematologic cancer (e.g. chronic lymphoid leukemia, myelodysplastic syndromes) may require ongoing maintenance therapy or life-long follow-up (“watchful waiting”) [4, 5].

### Elderly hematologic cancer patients

The older age is characterized by the increase of frailty, physical comorbidities, functional limitations, cognitive deficits, and inability to perform activities of daily living [7–9]. In elderly cancer patients, there is a significant correlation between somatic diseases, functional limitations and psychological distress [9–11]. Anxiety and depression are considered the most common forms of psychological distress in elderly cancer patients with the highest prevalence in patients older than 80 years [7, 10, 11]. Furthermore, desease- and treatment-related symptoms and the awareness of living with an incurable malignancy can profoundly impact health-related quality of life [12].

In contrast to the amount of research available for patients with solid tumors, there is still a paucity of studies regarding health-related quality of life (HRQOL) in patients with hematologic malignancies [13–16]. For elderly hematologic cancer patients, the number of studies is even sparser. Although about every second cancer patient is at least 65 years of age [17], elderly patients are often not accorded access to treatment trials [18, 19]. Even if there is a growing interest of the haematology community in patient-reported outcomes, long-term data on quality of life remain limited [20, 21]. Consequently, little is known about drug interactions when both cancer therapy *and* treatment for comorbid conditions are carried out at the same time. Knowledge is lacking about social and psychological states of older cancer patients undergoing cancer treatment and their cognitive and functional status. Therefore, treatment decisions may not be evidence-based and supportive care needs may be left unmet [22]. Elderly patients with severe functional or cognitive deficits will presumably have difficulties participating in complex treatment regimens and, thus, treatment adherence might be adversely affected [23, 24]. Furthermore, in the treatment of elderly hematologic patients considerations of expected beneficial and harmful effects of treatment, survival, and HRQOL gain importance [13].

Two studies focusing on psychosocial issues in elderly patients with hematologic cancer have reported the following results: According to a Canadian study with patients suffering from acute leukemia, old age is associated with higher levels of hopelessness and depression is associated with a higher burden of physical symptoms [25]. An Australian study with over 500 hematologic cancer patients reported lower perceived quality of “respectful communication” and “cancer information” in patients 40 years or older compared to adolescents and young adults (18–39 years). However, to develop and implement survivorship care plans and improve access and utilization of psychooncological support offers, it is necessary to improve the knowledge about psychological and physical symptom burden and specific supportive care needs in elderly hematologic cancer patients [26].

### Objectives

In view of previous research, the aims of the study are to assess:

- the prevalence of chronic conditions, functional disabilities and psychological distress in both elderly hematologic cancer patients aged ≥70 years and an age- and sex-matched sample of the general population;
- health related quality of life and social support in both samples;
- resources and risk factors having an impact on health related quality of life and psychological distress;
- perceived need for professional psychosocial support and utilization of support offers;
- patient satisfaction with information about the cancer disease and cancer treatments.

The findings should make an important contribution to the field of psychooncological care in elderly patients. Our study will provide both a data set offering detailed information about elderly hematologic cancer patients’ physical, psychological and social characteristics, and reference data of the elderly general population.

## Methods/design

### Study design

We conduct a prospective study with three measuring points: up to 5 years after first time diagnosis or diagnosis of relapse (t1), six months (t2) and one year after t1 (t3). The time after diagnosis was limited to five years in order to include short- and mid-term cancer survivors and, thus, to limit the heterogeneity of the study population. In addition, a cross sectional study with a sample of the general population matched by age and sex has been undertaken to compare psychological distress, chronic conditions and quality of life in patients completing cancer treatments and the elderly population.

## Study participants

### Inclusion criteria

Study participants (both patients and general population) need to be ≥70 years of age and have to give written informed consent.

Patients participating at the study must have a confirmed diagnosis of a hematologic cancer (ICD: C81 – C96) up to 5 years after primary diagnosis or relapse; and provide written informed consent.

### Exclusion criteria

Exclusion criteria comprise major communication difficulties and severe cognitive impairment that would interfere with an individual’s ability to give informed consent for research and complete study questionnaires.

## Recruitment and data collection

Written informed consent is obtained from all study participants prior to study participation. The study was approved by the Ethics Committee of the University of Leipzig, Faculty of Medicine (approval no. 071–14-10,032,014) and has therefore been performed in accordance with ethical standards.

The Cancer Registry of Leipzig (German city with about 540,000 inhabitants, of which about 91.000 are at least 70 years) provided names, addresses, birthdate, sex, ICD-10 diagnosis and date of first time diagnosis or relapse diagnosis of all cancer patients with a minimum age of 70 years. According to the Hospital Law of the German Federal State of Sachsen (§ 34 SächsKHG) patients’ agreement is required for the dissemination of patient-specific information. Thus, only patients treated in hospitals routinely requiring such an agreement before admission were eligible for study participation. Patients eligible for study enrollment were treated in three Leipzig based hospitals: University Medical Center Leipzig and two general hospitals. Patients treated in University Medical Center Leipzig, but not being registered in the Cancer Registry because they lived outside the Leipzig area, were identified using the hospital’s patient database.

We started patient recruitment in August 2014. While the t1 interviews were completed in 2016 May, follow-up interviews will be accomplished in December 2016 (t2) and May 2017 (t3) respectively. Patients eligible for study participation received an invitation letter informing about the study and a stamped postcard giving the options to either agree with or refuse study participation. Patients who agreed to participate received a phone call in order to make an appointment for an interview. Patients who did not wish to be personally interviewed were mailed a questionnaire. Study participants were offered an incentive of 10 Euro. Patients who had not responded after three weeks where mailed another letter asking them once more to take part at the study.

The population survey was conducted between March and October 2015. The local residents’ registration office of the German city of Leipzig provided names, addresses, age and sex for a sample of 600 persons with a minimum age of 70 years. In Germany, local residents’ registration offices are permitted to disseminate contact data (addresses) of all local residents to scientific institutions. Persons received an invitation letter informing about the study, the questionnaire and a stamped return envelope. Study participants were offered an incentive of 10 €. Persons who did not respond after three weeks were mailed another letter asking them once more to answer the questionnaire.

## Study measures

### Demographics, medical history and functional performance

#### Demographics

Demographic questions include age, sex, marital and partnership status, number of children, education level, size of household, monthly household income, nationality and religious affiliation.

#### Chronic conditions

To assess the prevalence and burden of chronic conditions, we employ a modified version of a self-report instrument developed by Bayliss et al. [27]. The original instrument comprises a list of 23 common chronic medical conditions, of which 17 were included in the modified instrument. Six conditions were excluded from the list, because they were either assessed with other instruments (overweight, hard of hearing) or seemed not appropriate for self-assessment (poor circulation, elevated cholesterol, angina/coronary artery disease, osteoarthritis). Two further conditions were added: psychological conditions and sensation disorders (tingling, numbness, polyneuropathy). Thus, our list comprises the following conditions.

Hypertension, asthma, chronic Bronchitis/COPD, diabetes, thyroid disorder, back pain, rheumatic disease, rheumatoid arthritis, osteoporosis, colon problems (e.g., diverticulitis, irritable bowel), stomach problems (e.g., gastritis, peptic disease), kidney disease, congestive heart failure, stroke, nerve condition (e.g. Parkinson’s disease, multiple sclerosis), (secondary) cancer, vision problems, psychological conditions and sensation disorders.

To assess the prevalence of these conditions, respondents are asked whether they have these conditions (dichotomous answer). To assess the burden of chronic conditions, respondents who have the condition are also asked to specify how the condition interferes with their daily activities. The items are scored on a 5-point Likert scale ranging from 1 (not at all) to 5 (a lot). Based on the 19 items, a total score representing the level of morbidity can be computed. The total score ranges between 0 and 95 and represents the sum of conditions weighted by the level of interference assigned to each condition [28].

#### Geriatric screening

The geriatric screening instrument used in our study was developed by Lachs et al. and aims to identify common functional disabilities in elderly patients [29]. It comprises tests of vision, hearing, arm and leg function, urinary incontinence, mental status, over- or underweight, instrumental and basic activities of daily living, environmental hazards, and social support systems [29]. The instrument consists of 15 binary-choice items that must be answered with either “yes” (value 1) or “no” (value 0). Based on the items a total sum score may be computed with higher values representing a worse functional status.

### Psychological distress

#### Patient health questionnaire (PHQ-9)

The PHQ-9 is the nine-item depression module of the “Patient Health Questionnaire” [30]. Each of the items is scored on a 4-point Likert scale ranging from 0 (not at all) to 3 (nearly every day). The items are summed to obtain a total score ranging from 0 to 27 with higher scores indicating greater severity of depression [31]. Based on the total score, different levels of severity can be evaluated with 0–4, 5–9, 10–14, and 15–27 points indicating “minimal”, “mild”, “moderate”, and “severe” depression [31]. Because of its excellent criterion validity and sensitivity to change, the PHQ-9 can be used for longitudinal as well as for cross-sectional studies [32].

#### General anxiety disorder-scale (GAD-7)

The GAD-7 is a seven-item screening instrument and severity measure for Generalized Anxiety Disorder (GAD) with good reliability as well as criterion and construct validity [33]. Similar to the PHQ-9 questionnaire, each of the GAD-7 items is scored on a 4-point Likert scale ranging from 0 (not at all) to 3 (nearly every day). The items are summed to create a total score (range from 0 to 21) with higher scores indicating greater severity of anxiety. Based on the total score, different levels of severity can be evaluated with 0–4, 5–9, 10–14, and 15–21 points indicating “minimal”, “mild”, “moderate”, and “severe” GAD [33].

#### NCCN distress thermometer

The NCCN distress thermometer is a screening tool developed by the American “National Comprehensive Cancer Network” (NCCN) to assess psychological distress [34]. It comprises a visual-analogue scale to assess the severity of psychological distress and a problem list to identify patient needs. In the current study, we used the scale only. It ranges from 0 to 10 with higher values indicating more severe distress. A score ≥ 5 is considered an indicator that a patient is distressed and needs support [35].

#### Geriatric depression scale short version-15 (GDS-15)

The Geriatric Depression Scale (GDS) is one of the most widely used screening instruments for depression in elderly patients [36]. The GDS-15 short form used in the current study comprises 15 binary-choice items that must be answered with either “yes” (value 1) or “no” (value 0) [37]. The items are summed to produce a total score ranging from 0 to 15 with higher scores indicating greater severity of depression. Based on this score, three levels of depression severity can be derived: 0–5, 6–10, and 11–15 standing for “mild”, “moderate”, and “severe” depression [36].

### Health-related quality of life

#### EORTC quality of life questionnaire (EORTC QLQ-C30)

The EORTC QLQ-C30 is the core questionnaire of the “European Organisation for Research and Treatment of Cancer” for evaluating health-related quality of life. It is a 30-item instrument comprised of five functioning scales, nine symptom scales and one scale measuring “Global quality of life” (GQoL). All scales have a score range between 0 and 100. While high scores of the symptom scales indicate a high burden of symptoms, high scores of the functioning scales and on the GQoL scale indicate better functioning resp. quality of life.

The EORTC QLQ-C30 has “satisfactory to excellent psychometric properties” [38]. High reliability (Cronbach’s alpha >0.70) and good construct validity were also confirmed for all scales of the German version [39].

#### EORTC elderly cancer patients questionnaire (EORTC QLQ-ELD14)

The 14-item EORTC QLQ-ELD14 questionnaire is an EORTC module addressing HRQOL in elderly cancer patients. It comprised of five multi- and two single-item scales addressing the following age-specific issues: mobility, joint stiffness, worries about others, future worries, burden of illness, maintaining autonomy and purpose, and family support. Each scale has a score range between 0 and 100. While on the first four of these scales, higher scores indicate poorer mobility, more severe joint stiffness, higher degrees of worries about others and future worries, and a higher burden of illness, on the last two of these scales, higher scores stand for better family support and maintenance of autonomy and purpose respectively [40].

#### Brief fatigue inventory (BFI)

The Brief Fatigue Inventory (BFI) is a ten-item screening tool to assess severity of fatigue and fatigue-related impairment in cancer patients [41]. The BFI starts with a binary-choice question asking the respondent whether he feels more than usual fatigued or tired. The initial question is followed by nine eleven-step numerical rating scales measuring the intensity of fatigue (3 items) and the impairment in several domains related to fatigue (6 items). The rating scales have a score range between 0 and 10 with higher scores signifying higher intensity and impairment. The German version of the BFI was found to be reliable and valid [41].

#### Questions on life satisfaction (FLZ)

The questionnaire “Questions on Life Satisfaction” is an instrument for assessing general and health-related quality of life. It comprises two modules: The General Life Satisfaction module (“General LS”) and the Satisfaction with Health module (“Health”). In the current study, only the General LS module is used. The module covers eight relevant areas of life, that is: friends/acquaintances, leisure time/hobbies, health, income/financial security, occupation/work, housing/living conditions, family life/children, and partner relationship/sexuality (15). In a first step, the respondents are asked to rate the importance of these areas of life for their overall satisfaction. In a second step, the respondents are asked about their degree of satisfaction in each of these areas. The responses are given on a five-point Likert scale ranging from 0 (not important/not satisfied) to 4 (extremely important/very satisfied). Based on both the ratings of importance and satisfaction, a total score yielding information about “weighted satisfaction” can be computed. The total score ranges between −12 and +20 with higher scores reflecting a higher degree of satisfaction [42].

#### EORTC information questionnaire (EORTC QLQ-INF25)

The EORTC QLQ-INF25 is the information module of the “European Organisation for Research and Treatment of Cancer” to evaluate the information received by cancer patients. The module comprises 25 items, 21 of which are scored on a 4-point Likert scale ranging from 1 (not at all) to 4 (very much) and 4 of which are binary-choice questions that must be answered with either “yes” (value 1) or “no” (value 0). Based on the items, 4 multi-item, 8 single-item scores and a global score can be calculated. Each scale has a score range between 0 and 100, with higher scores standing for having received more information [43].

### Social support

#### Lubben social network scale short version-6 (LSNS-6)

The Lubben Social Network Scale (LSNS) was developed to assess social integration and to screen for social isolation in elderly populations. Its short form comprises six items scored on a 6-point Likert scale ranging from 0 (no relatives/friends) to 5 (9 or more relatives/friends). Based on the items, two subscales (family and friends) each based on three items and a total scale based on six items can be calculated. While the subscales have a score range between 0 and 15, the total scale ranges from 0 to 30 with higher values indicating better social integration. In addition, cut-off points separating socially isolated from not socially isolated respondents can be computed. Based on the total score, respondents with a score of less than 12 can be identified as socially isolated. Based on the sub scores, respondents with a score of less than 6 are considred to have marginal family resp. friendship ties [44].

#### Illness-specific social support scale modified German short version (SSUK-8)

To assess social support we used a modified German short form of the 24-item “Illness-Specific Social Support Scale” [45]. The instrument (SSUK-8) aims to assess self-perceived social support in patients with chronical diseases. The SSUK-8 comprises eight items scored on a 5-point Likert scale ranging from 0 (never) to 4 (all the time). Each of the two subscales (“positive support” and “detrimental interaction”) is based on four items and has a score range between 0 and 16. While on the “positive support” subscale higher scores indicate better support, higher scores on the “detrimental interaction” subscale stand for more pronounced detrimental interaction between the patient and his or her significant others [46].

### Partnership

#### Partnership questionnaire short form (PFB-K)

The short form of the Partnership Questionnaire (Partnerschaftsfragebogen, PFB-K) is an instrument to assess satisfaction with and quality of partnership [47]. The instrument comprises nine items evaluating three dimensions of partnership: conflict behavior, tenderness, and togetherness/communication. The items are scored on a 4-point Likert scale ranging from 0 (never/very rarely) to 3 (very often). Based on these items, three sub scores ranging from 0 to 9 and a total sum score ranging from 0 to 27 with higher values reflecting better quality of partnership may be computed. One additional item scored on a 6-point Likert scale (very unhappy - very happy) is to assess happiness in partnership.

## Statistical methods

### Statistical analyses

Quantitative data analysis will be carried out using SPSS, GNU R and Microsoft Excel. Summary statistics will be calculated for both continuous (frequencies, mean, standard deviation) and categorical variables (frequencies, percentages). Comparisons between groups, e.g. patients and population, will be done using the chi-squared test (for categorical variables) and Student’s t-test for continuous independent samples. Longitudinal differences in means will be assessed using analysis of variance (ANOVA) with repeated measurements. The impact of relevant variables on quality of life and psychological distress will be evaluated using multiple linear and logistic regression models.

## Results

### Patient sample

**Fig. 1:**
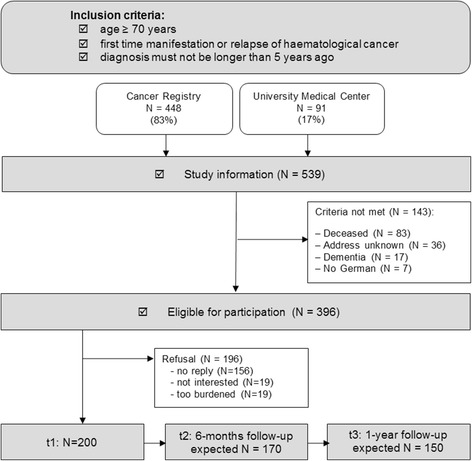
Study design and patient sample

In total, the Cancer Registry provided a list of 539 hematologic cancer patients. After excluding patients who had died (*n* = 83), had an unknown address (*n* = 36), had dementia (*n* = 17) or did not speak German (*n* = 7), 396 patients were eligible for study participation. 196 patients either refused to participate or did not respond. Thus, with 200 participants the overall participation rate was 50.5% (Fig. 1).

### Population sample

**Fig. 2:**
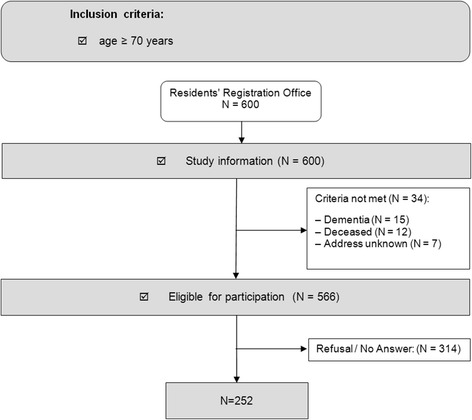
Study design and population sample

After excluding persons who had dementia (*N* = 15), had already died (*N* = 12) or had an unknown address (*N* = 7), 566 persons were eligible for study participation. 314 of these persons either refused to participate or did not respond. Thus, with 252 participants the overall participation rate was 44.5% (Fig. 2).

## Demographic and clinical characteristics

Nearly 2/3 of both patient and population sample were male. With a mean age of 76.2 years, the patient sample (t1) was significantly younger than the population sample. However, the age difference between both samples was rather small (1.2 years). While about 57 vs. 41% were younger than 75 years (patients vs. population), about 18 vs. 25% were older than 80 years (patients vs. population). There were no statistical significant differences between both samples regarding marital and partnership status. The majority of participants in both samples was married or cohabiting. About nine in ten study participants in both samples had one or more children. With 36 vs. 45% (patients vs. population), a significantly larger part of the population sample had a higher education level. In both samples, fewer than three in ten study participants were member of a religious group or church. There were no significant differences between both samples regarding monthly net household income. While about 17 vs. 7% had a Care level (nursing care), 41 vs. 24% had a Degree of disability (patients vs. population). Both differences were statistically significant (Table 1).

**Table 1:** Demographic sample characteristics (t1)

	Patients	Population	*p*
n	200	252	
Age (mean (SD))	76.19 (4.91)	77.54 (4.84)	0.003
Age groups	n (%)	n (%)	0.006
70–75 yrs	114 (57.0)	102 (40.5)	
76–80 yrs	51 (25.5)	88 (34.9)	
81–85 yrs	25 (12.5)	41 (16.3)	
> 85 yrs	10 (5.0)	21 (8.3)	
Gender: male	128 (64.0)	153 (60.7)	0.537
Marital status			0.488
married	144 (72.0)	172 (68.3)	
single	7 (3.5)	14 (5.6)	
divorced	12 (6.0)	17 (6.7)	
widowed	37 (18.5)	49 (19.4)	
Partnership: yes	150 (75.0)	174 (69.0)	0.197
Children: yes	184 (92.0)	221 (87.7)	0.183
Education level			0.040
8 yrs	85 (42.5)	103 (40.9)	
10 yrs	43 (21.5)	35 (13.9)	
High school certificate	4 (2.0)	14 (5.6)	
university	68 (34.0)	100 (39.7)	
Net household income			0.819
<1000€	18 (9.0)	21 (8.3)	
<2000€	108 (54.0)	127 (50.4)	
<3000€	60 (30.0)	89 (35.3)	
≥3000€	9 (4.5)	9 (3.6)	
missing	5 (2.5)	6 (2.4)	
Religion			0.950
none	150 (75.0)	185 (73.4)	
protestant	42 (21.0)	58 (23.0)	
catholic	4 (2.0)	5 (2.0)	
other	4 (2.0)	4 (1.6)	
Nursing Care level: yes	34 (17.0)	17 (6.7)	0.001
Degree of disability: yes	81 (40.5)	60 (23.8)	<0.001
Years since diagnosis (mean (SD))	2.6 (2.4)	NA	

In the patient sample, the diagnoses were as follows: lymphoma (*N* = 103, 52%), leukemia (*N* = 54, 27%), and myeloma (*N* = 43, 22%) (Table 2).

**Table 2:** Clinical sample characteristics (t1)

ICD-10	Name	n	%
C81	Hodgkin lymphoma	2	1
C82	Follicular lymphoma	19	10
C83	Non-follicular lymphoma	39	20
C84	Mature T/NK-cell lymphomas	17	9
C85	Other and unspecified types of non-Hodgkin lymphoma	20	10
C88	Malignant immunoproliferative diseases	5	3
C90	Multiple myeloma and malignant plasma cell neoplasms	43	22
C91	Lymphoid leukaemia	20	10
C92	Myeloid leukaemia	29	15
C93	Monocytic leukaemia	2	1
C94	Other leukaemias of specified cell type	1	1
C96	Other and unspecified malignant neoplasms of lymphoid, haematopoietic and related tissue	3	2

While 20 patients (10%) did not receive any cancer therapy (not in table), more than three in four patients either had received or were receiving chemotherapy. Radiotherapy and transplantation were received by 31% and 16% and miscellaneous therapies (e.g. antibody, immunosuppressive therapies) by 26% of patients (Table 3).

**Table 3:** Hematologic cancer therapies (t1)

	Completed		Running	
	n	%	n	%
Chemotherapy	107	54	47	24
Radiotherapy	59	30	1	1
Transplantation	33	16	0	0
Misc. therapies	31	16	19	10
Total	117	58	63	32

## Non-responder analysis

In the patient sample, study participants were younger than patients who refused participation (not in table). Age differences between both groups were statistically significant (t-test, *t* = 2.82, df = 392, *p* = 0.005) but rather small (1.4 years). With participation rates of 57.5% and 41.8% for male and female patients, there were significant differences between both sexes regarding study participation (*Chi* ^2^=9.07, *p* = 0.003).

In the population sample, study participants were younger than persons who refused participation (not in table). Age differences between both groups were statistically significant (t-test, *t* = 2.29, df = 550, *p* = 0.022) but small (1 year). There were no significant differences in the participation rates of women and men (*Chi* ^2^=0.16, *p* = 0.69).

## Discussion

The primary objectives of this prospective study are to assess the prevalence of chronic conditions, functional disabilities, mental disorders and psychological distress in both elderly hematologic cancer patients and an age-matched sample of the general population, and to assess health related quality of life and social support in both samples respectively. We have enrolled both a sample of 200 hematologic cancer patients and a sample of the general population (*N* = 252). Follow-ups will be completed in May 2017. Compared to the data published by the Robert-Koch-Institut, male patients and patients with myeloma are slightly overrepresented in our sample, whereas female patients and patients with leukemia are underrepresented. Furthermore, there are significant but small differences between patient and population sample regarding age. Thus, caution must be exercised in the interpretation of the study results.

In summary, our study will provide both a data set offering detailed information about elderly hematologic cancer patients’ physical, psychological and social characteristics, and reference data of the elderly general population. Thus, we will be able to investigate to which extend the patients’ burden of disease is age-related and to which extend it must be attributed to cancer and cancer treatment.

Furthermore, the study will provide important information for the development and implementation of psychooncological support offers and survivorship care plans.

## Abbreviations

ANOVA: Analysis of variance
BFI: Brief Fatigue Inventory
EORTC QLQ-C30: Quality of life core questionnaire of the European Organisation for Research and Treatment of Cancer
EORTC QLQ-ELD14: Quality of life questionnaire of the European Organisation for Research and Treatment of Cancer, module for elderly cancer patients
EORTC QLQ-INF25: Quality of life questionnaire of the European Organisation for Research and Treatment of Cancer, information module
FLZ: Questions on Life Satisfaction
GAD-7: General Anxiety Disorder scale
GDS-15: Geriatric Depression Scale short version
LSNS: Lubben Social Network Scale short version
NCCN: National Comprehensive Cancer Network
PFB-K: Partnership Questionnaire short form (PFB-K)
PHQ-9: Patient Health Questionnaire
SD: standard deviation
SSUK: Illness-Specific Social Support Scale modified German short version
